# HIV-Enhancing and HIV-Inhibiting Properties of Cationic Peptides and Proteins

**DOI:** 10.3390/v9050108

**Published:** 2017-05-15

**Authors:** Alexander M. Cole, Amy L. Cole

**Affiliations:** Division of Molecular Microbiology, Burnett School of Biomedical Sciences, UCF College of Medicine, 4110 Libra Drive, Orlando, FL 32816, USA; amy.cole@ucf.edu

**Keywords:** HIV, antiviral, viral enhancer, cationic, antimicrobial, polypeptide

## Abstract

Cationic antimicrobial peptides and proteins have historically been ascribed roles in innate immunity that infer killing of microbial and viral pathogens and protection of the host. In the context of sexually transmitted HIV-1, we take an unconventional approach that questions this paradigm. It is becoming increasingly apparent that many of the cationic polypeptides present in the human genital or anorectal mucosa, or human semen, are capable of enhancing HIV-1 infection, often in addition to other reported roles as viral inhibitors. We explore how the in vivo environment may select for or against the HIV-enhancing aspects of these cationic polypeptides by focusing on biological relevance. We stress that the distinction between enhancing and inhibiting HIV-1 infection is not mutually exclusive to specific classes of cationic polypeptides. Understanding how virally enhancing peptides and proteins act to promote sexual transmission of HIV-1 would be important for the design of topical microbicides, mucosal vaccines, and other preventative measures.

## 1. Introduction

In 2015, there were an estimated 36.7 million people living with HIV worldwide, 1.1 million people died from HIV-related causes globally, and the cumulative death toll had risen to more than 35 million people [1]. Subsaharan Africa was the most affected region globally, accounting for over two-thirds of the people living with HIV as well as two-thirds of new HIV infections. In the same year, an estimated 1.2 million people were living with HIV in the U.S., and approximately an eighth of these individuals are estimated to be unaware of their infection status [2]. Over the decade from 2005 until 2014, the number of new HIV diagnoses in the USA had fallen 19%. Due to increased HIV testing, improved availability of antiretroviral therapy, and emerging antiretroviral prophylaxes, this suggests that there had been a tangible decline in HIV infections during that time period. While the epidemic appears to be stabilizing in the USA, this perception has given rise to an increasing level of complacency in individuals who view HIV as a clinically manageable chronic condition. This recent, disturbing trend is compounded by the fact that, with an increasing number of people receiving antiretroviral therapy, viral resistance to most of the available drugs is inevitable. In order to continue controlling the pandemic, there must be a constant influx of next-generation antiretrovirals coupled with other innovative strategies to combat HIV. 

The holy grail of HIV prevention would be an effective, long-term HIV vaccine, yet vaccine trials to date have shown little to no promise in the clinic [3]. In the absence of an available vaccine, the field has turned to various methods to prevent or limit the transmission of HIV. The most successful prevention strategy to date, termed Pre-Exposure Prophylaxes (PrEP), has utilized oral antiretrovirals to treat the uninfected partner of a serodiscordant couple prophylactically, to reduce the incidence of HIV transmission from the HIV infected partner [4]. It should also be noted that this and other oral PrEP studies have shown greater efficacy in treating the infected partner, underscoring the importance of limiting viral load in an infected individual to reduce the likelihood of sexual transmission to the uninfected partner. However, as with any antiretroviral therapy given to an infected individual, the likelihood of HIV acquiring resistance would remain high. 

Other promising prevention strategies studied over the past two decades include topical microbicides, which are prophylaxes self-administered by an uninfected individual to the cervix/vagina or anus/rectum to prevent or limit sexual or vertical (mother to newborn) transmission of HIV. This type of prevention therapy targets these highly HIV-susceptible mucosal environments, and has several potential benefits: (1) surreptitiously applying a gel, cream, film, or other slow-release method to provide long-lasting protection could empower individuals from societally vulnerable populations to take HIV prevention measures in their own hands; (2) the topical application of a microbicide is confined to surfaces of the body where sexual or vertical (mother to newborn) transmission of HIV is greatest, vastly reducing the amount of drug required as compared to systemic delivery; (3) targeting prevention measures to the uninfected partner should substantially reduce the likelihood of HIV resistance; and (4) formulations engineered to retain the antiretroviral drug in the mucosa and reduce systemic absorption could also reduce or delay the onset of viral resistance. 

As with HIV vaccines, an all-too-familiar lack of success has plagued the field of topical microbicides. While self-applied prophylaxes have the potential to target populations that are susceptible to acquiring HIV sexually or vertically, clinical trials have instead revealed that patient acceptability, psychosocial issues, and adherence to proper drug application regimens were more problematic than originally envisioned and likely contributed to failures in the clinic [5]. The rapid emergence of the new field of topical microbicides led to questionable early decisions to promote compounds, which were insufficiently vetted prior to trials in the clinic. Early iterations of unsuccessful topical microbicides included nonspecific molecules that either did not prevent the transmission of HIV-1 (Carraguard, cellulose sulfate, PRO2000, Cyanoviran), or increased the likelihood of infection due to perturbations to the cervicovaginal or anorectal mucosa (e.g., nonoxynol 9) [6]. While some could point to a number of concerns raised, which may have been disregarded at the time, others might argue that the field now has the benefit of hindsight, and that the problems encountered drove the development of next-generation compounds and formulations [6]. It is probably a mix of both. Irrespective of the reason, these early failures tainted the reputation of topical microbicides, and the field has had an uphill battle to develop next-generation topical preventatives while simultaneously attempting to suppress the stigma that microbicides are ineffective. More recent topical microbicide trials have employed formulations of known antiretroviral drugs, such as the nucleotide analog reverse transcriptase inhibitor tenofovir, with questionable success [5].

Perhaps more instrumental, other factors have contributed to the lack of success of topical microbicides, including biocompatibility with the unique environments of the cervicovaginal and anorectal mucosa [7,8]. This area has recently gained traction, since developing an effective topical microbicide, or a mucosal vaccine, would require an extensive appreciation of the local mucosal environment and how the preventative treatment would interact with that surface. The cellular composition and architecture of each mucosa are amongst many topics that would be important to consider when developing topical microbicides and mucosal vaccines. The bulk of this review is focused on a subset of those aspects, specifically cationic peptides and proteins located within mucosal secretions, which are produced by the respective epithelia and immune cells. These molecules are commonly termed antimicrobial polypeptides (AMPs) or host defense polypeptides (HDPs), due to their reported wide spectrum of activity against viruses, bacteria, and fungi [9] or broader roles in innate immunity [10]. 

The descriptions of AMPs and HDPs imply a predominantly host-protective role of these peptides and proteins. In the paradigm that regards microbes and viruses as villainous, this historical viewpoint tends to skew perception that AMPs and HDPs are present solely to help rather than harm the host, akin to naturally produced antibiotics. Instead, the converse reality is that a number of cationic peptides and proteins reportedly act by promoting or enhancing microbial or viral infection. Yet, even the recent literature is predominated by far more articles that use terms for polypeptides that kill microbes or limit their infection than polypeptides that promote or enhance infection. Why does the host-benefitting paradigm still predominate? One reason could be that the field is entrenched due to a funding environment that disproportionally favors the discovery of novel antibiotics and new pro-immune standards. Another possibility is that “what’s past is prologue,” seeking what we expect to discover and ultimately finding what we have set out to seek. Thus, groups that historically have studied antimicrobial polypeptides are likely to uncover similar molecules, pathways, and processes largely because their hypotheses, experimental designs, and methodologies are often geared in that manner. 

We take a somewhat uncommon tack, focusing on the dichotomous relationship that cationic peptides and proteins have with regard to promoting or preventing HIV-1 transmission and infection. Background on the sexual transmission of HIV-1 or its intracellular lifecycle are not covered due to space constraints; instead, the reader is referred to a comprehensive review on this subject [11]. We explore how the in vivo environment may select for or against the HIV-enhancing aspects of these cationic polypeptides by focusing on biological relevance: all molecules presented have been identified in pertinent mucosa that are the main tissue targets for HIV-1 transmission, or in the fluids that overlie those mucosal surfaces. We stress that the distinction between enhancing and inhibiting viral infection is not mutually exclusive to specific classes of cationic peptides or proteins. Understanding how virally enhancing peptides and proteins act to promote sexual transmission of HIV-1 in the mucosa may help interpret past failures in the clinic, and would be important for the design of future topical microbicides and mucosal vaccines. 

## 2. Defensins

Defensins are the first family of cationic antimicrobial peptides to have been discovered in human cells [12,13]. The three classes of defensins are categorized primarily by the disulfide bonding pattern of their six cysteine residues: α-defensins, β-defensins, and θ-defensins [14]. In humans, α-defensins and β-defensins are produced as mature peptides by myeloid cells and epithelia. Only select nonhuman primates produce θ-defensins, since θ-defensin genes in humans are only transcribed into mRNA [15,16]. Under conditions tested, the translation of θ-defensin peptides is prevented by a premature termination codon present in the putative signal sequence [15,16].

The two main types of human α-defensins are human neutrophil peptides 1–4 (HNP1–4) and human defensins 5 and 6 (HD5, HD6). Each type of α-defensin has been reported to exhibit disparate actions in preventing and/or promoting HIV-1 infection. Azurophil granules in human neutrophils contain extraordinarily large amounts of mature, processed HNPs, which comprise approximately one third of the granules’ protein content and can reach the millimolar concentration range [17,18,19]. α-defensins were the first anti-HIV-1 peptides discovered, when Nakashima and colleagues described that α-defensins from a variety of small mammals inhibited HIV-1 replication and reduced associated T cell cytopathology [20]. Human HNP1 is also active against HIV-1, acting by directly targeting HIV-1 as well as interfering with protein kinase C [21]. Structural similarities between a looped region of the gp41 glycoprotein from the envelope of HIV-1 and α-defensins pointed to an antiviral mechanism that prevented the entry of virus into target cells [22]. Since HNPs are produced and stored in neutrophils, unless clinical or subclinical inflammation exists in the anogenital mucosa, the concentrations of these peptides are quite low. Even at concentrations required for modest anti-HIV-1 activity in vitro (i.e., tens of micrograms/mL), HNPs are cytotoxic to human cells and further enhance localized tissue inflammation [23]. The physiological relevance of α-defensins as antiretroviral agents in the anogenital mucosa is therefore suspect. Does increased inflammation, and associated recruitment of CD4+ cellular targets for HIV-1, outweigh the intrinsic antiretroviral activity of these peptides? A study by Levinson and colleagues supports this assertion, revealing that higher levels of two primarily neutrophil-derived cationic antimicrobial peptides, HNPs and LL-37, are associated with genital infections and increased acquisition of HIV [24].

HD5 and HD6 were originally isolated from Paneth cells located at the base of the Crypts of Lieberkühn within the small intestine [25,26,27], and are among the historically defined broad-spectrum antimicrobial peptides. Unlike HNPs, which are stored in their mature form, HD5 and HD6 are stored in secretory granules as inactive propeptides that are proteolytically activated by Paneth cell-derived trypsin upon granule release [28]. In vitro, HD5 and HD6 have been reported to augment HIV-1 infection, through the enhancement of viral attachment to target cells [29]. In the female reproductive tract, HD5 has been localized to ectocervical, endocervical and vaginal epithelia, and has been found specifically within granules of endocervical epithelium [30]. HD5 is maximally expressed during the uterine secretory phase of the menstrual cycle [30], potentially providing a window of opportunity for increased susceptibility to HIV-1 transmission and infection. As one insight into the clinical complications observed with topical microbicides, HD5 and HD6 antagonize the anti-HIV-1 activity of polyanion-based topical microbicides, likely reducing their prophylactic benefit in vivo [31]. The cervix is the principal site whereby HIV-1 initiates infection in the female reproductive tract [32,33,34]. HD5 and HD6 expressed by cervical epithelia may therefore be critical for helping establish HIV-1 infection in this tissue. Conversely, another group reported that HD5 inhibits HIV-1 infection [35], utilizing serum-free and low-ionic strength conditions in an attempt to mimic infection in mucosal fluids. The anti-HIV-1 activity of HD5 is likely artifact, since under serum-deprived conditions, HD5 blocked CD4 receptor-independent HIV-1 infection, attributable to an HD5-mediated increase in cell death in primary CD4+ T cells rather than a specific antiretroviral mechanism [36]. In support of the HIV-enhancing role for HD5, individuals with sexually transmitted infections and bacterial vaginosis, both of which are associated with increased risks of HIV transmission, also had elevated levels of HD5 and other defensins [37,38]. 

In humans and certain nonhuman primates, θ-defensin genes appear to be evolutionary descendants of α-defensins [15,39]. Although it is known that mature, 18 residue θ-defensins from nonhuman primates are the amalgamation of two smaller gene products [39], the mechanisms involved in their splicing, folding and macrocyclization remain mostly unresolved [40]. Human θ-defensins are transcribed into mRNA [15,16], yet the premature termination codon precludes translation into peptides under normal conditions. This has sparked curiosity about why these expressed pseudogenes have remained otherwise intact since the evolutionary timepoint when humans diverged from orangutans—the last known human ancestor that expresses both intact and prematurely terminated θ-defensins [16]. Moreover, human θ-defensins retain nearly 90% identity at the nucleotide level with θ-defensins from rhesus macaques [16], a level high enough to warrant speculation about contemporary functions for these genes. Several attempts have been made to answer this question. Human θ-defensin peptides called “retrocyclins” were recreated using solid-phase synthetic approaches, based on genetic information retained in their intact genes. Synthetic retrocyclins are reportedly very active against most lab-adapted strains and clinical isolates of HIV-1 tested [15,41,42], acting through the inhibition of viral fusion and subsequent entry into target CD4+ cells [43,44]. In another study, partial suppression of the premature termination codon could restore the production of a modest level of intact retrocyclin peptides, which were active against HIV-1, from vaginal epithelia and promyelocytes [45]. This study also suggested that the complex molecular machinery required to produce retrocyclin peptides in humans remains at least partially functional, further supporting a contemporary function for these or other similarly processed peptides. A number of questions still remain, including whether the lack of functional retrocyclin peptides contributes to increased human susceptibility to HIV, whether retrocyclins were silenced evolutionarily as a result of undetermined adverse effects, and whether there are any natural conditions that could partially or completely suppress the premature termination codon to produce functional retrocyclins.

Human β-defensins (HBDs) are structurally distinct from α-defensins based on their disulfide connectivity, and are principally found in a variety of epithelia throughout the body [46]. Humans produce at least six β-defensin peptides, although only HBD2 and HBD3 are active in vitro against HIV-1 [47] at concentrations that generally exceed the normal physiological concentrations found in genital epithelial tissues or their overlying secretions. Initially, the primary antiretroviral mechanism of action for HBD2 and HBD3 was reported to downmodulate a chemokine coreceptor for HIV-1, CXCR4, one of two principal chemokine receptors utilized for viral entry into CD4+ cells [48]. Additionally, HBD3 had been shown to antagonize CXCR4 [49]. However, another group was unable to replicate these findings, instead implicating direct inactivation of HIV-1 virions and intracellular inhibition of HIV-1 replication [50]. Regardless, since most sexually transmitted HIV-1 utilizes CCR5 as a coreceptor for entry, a CXCR4-based mechanism would have limited function in the cervicovaginal mucosa. For HBD2, a more recent study has defined a post-entry mechanism of inhibition, due to the induction of CCR6-dependent APOBEC3G expression [51]. In vaginal fluid [52] and cervical mucus plugs [53], the concentration of HBD2 is present at nanograms/mL concentrations, which are far below the low micrograms/mL levels required for anti-HIV-1 activity in vitro [48]. Nevertheless, the physiological amounts of HBD2 in vaginal fluid are within the range reported to chemoattract immature dendritic cells and memory T cells in vitro, potential targets of HIV-1 [54,55,56]. In addition, HBD2 and HBD3 can chemoattract CCR2-expressing cells, including macrophages/monocytes and neutrophils [55], which can increase local tissue inflammation. Therefore, while HBD2 and HBD3 under normal physiological conditions unlikely contribute substantially to the direct anti-HIV-1 armamentarium, they may increase the incidence of viral transmission by augmenting inflammation and recruiting target cells for HIV-1. It should also be noted that localized concentrations of β-defensins might be appreciably higher at the surface of the mucosa, tilting the balance in favor of protection. Taken together, instead of only limiting the sexual transmission of HIV-1, the sum total of all defensins may place the anogenital mucosa under the dangling sword of Damocles. 

## 3. Semen-Derived Cationic Peptides

The most common mode of HIV-1 transmission to women is through exposure of the female reproductive tract to semen from HIV-infected men. Semen deserves particular attention in this review, since the literature is divided with regard to the potential of cationic polypeptides within this fluid to act as inhibitors or enhancers of HIV-1. Moreover, semen has been reported to impair the effectiveness of certain topical microbicides [57], underscoring the importance of assessing the efficacy of anti-HIV preventatives in the presence of this fluid. While semen is cytotoxic to target cells in vitro [58], similar to cervicovaginal fluid, the liquid component of human semen, seminal plasma, is intrinsically active against HIV-1 even at high dilutions in which cytotoxicity was not observed [59]. The bulk of this activity is reportedly due to cationic polypeptides, most of which are proteolytic fragments of several parent proteins [59]. From this study, a predominant semenogelin-derived peptide, termed “SG-1,” exhibited anti-HIV-1 activity in vitro at sub-physiological concentrations [59]. Additional fractionation of seminal plasma revealed a plethora of cationic polypeptides that could also inhibit HIV-1. While seminal plasma contains these and potentially other cationic antiviral components, other groups have demonstrated that certain cationic polypeptides within semen and seminal plasma can form fibrils that enhance HIV-1 infection in vitro [60].

A well-publicized study established that semen-mediated enhancement of HIV-1 transmission in vitro is due to cationic peptides derived from prostatic acid phosphatase (PAP), which aggregate to form amyloid fibrils called Semen-derived Enhancers of Viral Infection (SEVI) [60]. Further in vitro studies supported the role of SEVI in enhancing HIV-1 infection [61,62,63,64], which was due to the cationic properties of the PAP peptides [61]. These studies were put into question when another group revealed that the HIV-enhancing effects of SEVI may be diminished in vivo due to several semen-derived proteases that cleaved PAP peptides and prevented SEVI fibril formation [64]. A subsequent report emerged that countered this finding by suggesting that endogenous Zn^2+^ within seminal plasma might protect the fibrils from proteolysis [65]. Furthermore, SEVI fibrils were eventually detected in fresh human semen [66,67]. Anionic components such as inorganic phosphate and sodium bicarbonate within seminal plasma could also enhance SEVI fibril formation in vitro [65]. Most complex proteins have short, “sticky” segments that if exposed are capable of forming amyloid fibrils [68]. Indeed, under the right conditions, even cationic semenogelin fragments have also been shown to form amyloid fibrils that can enhance HIV-1 infection [69]. This suggested an alternative role for cationic semenogelin peptides and that multiple types of fibrils in semen may act in concert to promote HIV-1 infection. 

While the viral enhancing effects of SEVI and other similar fibrils have been extremely well documented in vitro, those findings have not translated in vivo. Intravaginally exposing rhesus macaques to increasing doses of the pathogenic SIVmac239 clone in the presence or absence of SEVI or seminal plasma did not reveal a significant difference in any of the experimental groups [70]. The first infections were at low viral doses, and the peak viral loads in those acutely infected macaques treated with SEVI or seminal plasma were approximately 6-fold higher than untreated animals [70]. Arguably, lower viral challenges might better represent natural infection conditions that would occur in vivo, and thus the definitive experiment to determine the effects of semen/SEVI in vivo is to conduct studies in a low-dose SHIV transmission model. In an effort to explain the in vivo findings, another group utilized female reproductive tract tissues from macaques and humans to understand the lack of SEVI-mediated viral enhancement by examining colocalization of fluorescent viral particles with stained SEVI fibrils [71]. SEVI reduced the number of HIV-1 virions penetrating stratified squamous epithelium, and in simple columnar epithelium, SEVI did not retain its fibrillar structure and became detached from the virions [71]. SEVI also did not enhance rectal HIV-1 transmission in two humanized mouse models [72]. Whether SEVI fibrils are simply not relevant in vivo, or antiretroviral cationic polypeptides from the cervicovaginal mucosa, rectal mucosa or seminal plasma are able to counteract the enhancing effects of fibrils remains unclear.

## 4. Other Cationic (Poly)Peptides in the Cervicovaginal Mucosa

Several other human cationic peptides and proteins have been reported to inhibit and/or enhance HIV-1 infection. hCAP18 is an 18 kDa protein in the cathelicidin family of antimicrobial peptides, which depending on the cellular environment, can be processed into at least three mature forms that differ by only one or two amino-terminal residues [73,74,75,76]. LL-37, the most widely studied, is stored in neutrophils and expressed by a variety of mucosal epithelia including the ectocervix in the female reproductive tract. Aside from antimicrobial activity, LL-37 is also immunomodulatory through the binding of N-formyl peptide receptor 2 (FPR2), a G protein-coupled receptor. LL-37 can antagonize FPR2, down-regulating chemokine coreceptors necessary for HIV-1 to bind primary CD4+ cells [77]. The concentration necessary to exert FPR2-mediated anti-HIV-1 activity in vitro [77] is within the range of LL-37 that is typically found in healthy vaginal fluid (mid-to-high nanograms/mL) [52]. On the other hand, herpes simplex virus (HSV)-2-infected keratinocytes produced LL-37 that increased the susceptibility of Langerhans cells to HIV-1 [78]. The HIV-1-enhancing effects could be neutralized by blocking LL-37 production [78]. The context of LL-37 production and interaction with other cellular and molecular entities are likely determinants that modulate this peptide’s activity toward HIV-1.

Neutrophils, epithelia and monocytes produce S100A8 and S100A9 proteins, the two subunits of calprotectin. While levels of calprotectin can account for over one-third of a neutrophil’s cytoplasm [79], in healthy vaginal fluid, the levels of calprotectin are present at low to mid micrograms/mL although higher concentrations have been identified in inflammatory conditions of the female reproductive tract. The subunit S100A8, isolated from cervicovaginal fluid, has been demonstrated to increase expression of HIV-1 in latently infected monocytes [80]. In contrast, S100A9 ligates CD85j on Natural Killer (NK) cells, increasing the activity of these cells against HIV-1 [81]. The serine protease Cathepsin G is another protein produced by neutrophils, which is present in cervicovaginal fluids [82]. While the exact mechanism is unclear, Cathepsin G binds gp120, an HIV-1 envelope glycoprotein, and enhances HIV-1 infection of macrophages [83,84,85]. Since the enhancement was not observed in purtussis toxin-treated macrophages, the mechanism likely involves Gi protein-mediated signal transduction [85]. The amino-terminus of RANTES, a natural ligand of the HIV-1 coreceptor CCR5, can also be proteolytically processed by Cathepsin G, rendering RANTES less active against HIV-1 [86]. Taken together, conditions that increase anogenital inflammation would serve to recruit cellular stores, and increase the local expression, of cationic peptides and proteins that increase susceptibility to HIV-1 infection. 

Two members of the whey acidic protein motif family reportedly modulate HIV-1 infection: secretory leukocyte protease inhibitor (SLPI) and trappin-2/elafin [87,88,89]. After more than two decades of research, it remains unclear as to whether SLPI is truly active against HIV-1. Under certain conditions, high nanomolar concentrations of SLPI could uncoat the capsid of HIV-1 in a protease-independent manner [90,91]. Although not related to its antiretroviral activity, women with sexually transmitted infections or bacterial vaginosis have reduced vaginal levels of SLPI, pointing toward the potential for increased transmission or infection by HIV-1 [92]. In stark contrast, another group determined that even milligram/mL concentrations of SLPI could not prevent HIV-1 infection [93]. It is possible that the primary utility of SLPI is as a biomarker of HIV-1-exposed seronegative individuals who appear more apt to limit infection [94]. The support for trappin-2/elafin as an anti-HIV-1 protein is more convincing [95]. Trappin-2/elafin targets the HIV-1 virion directly, and thus is active against both X4-tropic and R5-tropic strains. Epithelia of both the upper and lower female reproductive tract express constitutive levels of trappin-2/elafin, while only the epithelial cells in the uterus can be induced to express this protein [95]. Similar to HD5, trappin-2/elafin is likely hormonally regulated, with the highest levels present during the secretory phase of the menstrual cycle. Cervicovaginal lavage fluids from HIV-infected patients contain higher levels of trappin-2/elafin than uninfected patients [95], pointing toward HIV-1-induced regulation of this antiviral protein. 

Human lysozyme was the first cationic protein identified to have broad spectrum antibacterial activity [96,97]. In ocular and nasal fluids, the concentration of lysozyme can be extraordinarily high, reaching several milligrams/mL in healthy individuals [98,99]. By comparison, the level of lysozyme is in the low micromolar range in healthy cervicovaginal fluid [52], concentrations that are not anti-HIV-1 in vitro [82]. Due to its ability to bind HIV-1 RNA [100], lysozyme can lower the ability of HIV-1-infected primary T cells and monocytes to shed virus [101]; although the route by which this 14 kDa protein enters virions or infected cells is not evident. While full-length lysozyme is only modestly active against HIV-1, a nine-residue synthetic peptide engineered from a core region of lysozyme prevented HIV-1 entry at low to mid nanomolar concentrations [102]. While this nonapeptide has not been identified in human tissues or fluids, trypsin cleavage sites that flank this peptide suggest that proteolysis might occur in select physiological conditions. Lactoferrin is a component of neutrophil specific granules and is also produced by certain epithelia. Lactoferrin prevents HIV-1 entry into target cells by binding to the V3 loop of the HIV-1 envelope protein gp120 [103]. However, the level of lactoferrin is low in healthy vaginal fluid (low micromolar) [52], and since this protein is not active in vitro against HIV-1 at this physiologically relevant concentration [82], it is likely not effective in vivo or perhaps works in concert with other peptides and proteins. Over a dozen polypeptides related to host defense have been identified in the cervicovaginal fluids of healthy women, all of which are present at concentrations that individually cannot inhibit HIV-1 infection [82,104]. However in combination, the sum total of the cationic polypeptide components exhibited most of the anti-HIV-1 activity of cervicovaginal fluid, suggesting synergy between the different components [82]. 

## 5. Conclusions and Future Directions

As presented in this review, many classical cationic “antimicrobial” peptides and proteins are equally or more effective in enhancing rather than inhibiting HIV-1 infection. Since the discovery of cationic peptides and proteins, the field has promoted their potential as novel natural antibiotics that exert broad spectrum activity against a wide range of bacteria, viruses and fungi. It would have been difficult to establish this new field without touting the positive attributes of cationic polypeptides as templates for the design of next-generation therapeutic drugs. This aspect remains vitally important, as establishing a new class of antibiotics will be necessary to combat pathogens, which are becoming increasingly resistant to antimicrobial drugs currently available in the clinic. Yet with regard to host immune processes, it is now apparent that many cationic polypeptides are not protective or are potentially injurious to the host. It will be important to consider the effects of these polypeptides on immunity of the anogenital mucosa when developing topical microbicides and mucosal vaccines. The terms “antimicrobial peptides” and “host defense peptides,” which have defined the field, are becoming outdated and might be best reserved for particular subsets of molecules. We therefore conclude this review with a suggestion: rename the field to better encompass the role of cationic peptides and proteins, without prejudice. “Cationic Immunomodulatory Polypeptides” might be suitable.
